# Androgen receptor signaling and pyrethroids: Potential male infertility consequences

**DOI:** 10.3389/fcell.2023.1173575

**Published:** 2023-04-28

**Authors:** Ishfaq Ahmad Sheikh, Mohd Amin Beg, Taha Abo-AlmagdAbdel-Meguid Hamoda, Hammam Mahmoud Siraj Mandourah, Erdogan Memili

**Affiliations:** ^1^ King Fahd Medical Research Center, King Abdulaziz University, Jeddah, Saudi Arabia; ^2^ Department of Medical Laboratory Sciences, Faculty of Applied Medical Sciences, King Abdulaziz University, Jeddah, Saudi Arabia; ^3^ Department of Urology, King Abdulaziz University, Jeddah, Saudi Arabia; ^4^ College of Agriculture and Human Sciences, Prairie View A&M University, Prairie View, TX, United States

**Keywords:** androgen receptor, endocrine disruption, male infertility, pyrethroids, cypermethrin, deltamethrin, structural binding characterization

## Abstract

Infertility is a global health concern inflicting a considerable burden on the global economy and a severe socio-psychological impact. Approximately 15% of couples suffer from infertility globally, with a male factor contribution of approximately 50%. However, male infertility remains largely unexplored, as the burden of infertility is mostly assigned to female people. Endocrine-disrupting chemicals (EDCs) have been proposed as one of the factors causing male infertility. Pyrethroids represent an important class of EDCs, and numerous studies have associated pyrethroid exposure with impaired male reproductive function and development. Therefore, the present study investigated the potentially toxic effects of two common pyrethroids, cypermethrin and deltamethrin, on androgen receptor (AR) signaling. The structural binding characterization of cypermethrin and deltamethrin against the AR ligand-binding pocket was performed using Schrodinger’s induced fit docking (IFD) approach. Various parameters were estimated, such as binding interactions, binding energy, docking score, and IFD score. Furthermore, the AR native ligand, testosterone, was subjected to similar experiments against the AR ligand-binding pocket. The results revealed commonality in the amino acid-binding interactions and overlap in other structural parameters between the AR native ligand, testosterone, and the ligands, cypermethrin and deltamethrin. The estimated binding energy values of cypermethrin and deltamethrin were very high and close to those calculated for AR native ligand, testosterone. Taken together, the results of this study suggested potential disruption of AR signaling by cypermethrin and deltamethrin, which may result in androgen dysfunction and subsequent male infertility.

## 1 Introduction

Male infertility is a global health issue, with nearly 30 million male people suffering from this condition, contributing approximately 40%–50% to the overall couple infertility cases (Agarwal et al., 2015; Bold and Swinburne, 2022; Esteves et al., 2023; Wagner et al., 2023). Infertile men frequently experience a heavy psychological burden, such as anxiety, depression, trauma, stress, guilt, inadequacy, and personal distress, along with social issues, such as discrimination, ostracism, and divorce (Cui, 2010; Greil et al., 2011; Kumar and Singh, 2015; Lotti and Maggi, 2018; Afshani et al., 2020). In addition to the significant economic implications, infertility in men is increasingly becoming a marker of poor general health in affected men (Lotti and Maggi, 2018; De Jonge and Barratt, 2019). The available literature indicates that for the past several decades, male reproductive health has globally shown a declining trend with a decrease in average serum testosterone levels and semen parameters, such as sperm concentrations and semen volume, along with an increase in the incidence of congenital cryptorchidism and testicular tumors (Rodprasert et al., 2021). In this regard, the causes of poor semen quality in approximately 45% of cases of infertile men are idiopathic with no known reasons (De Jonge and Barrat, 2019; Alahmar et al., 2022). However, in general, the majority of the available studies on infertility are focused on women because of the onus of the burden of infertility on them. Hence, male aspects of infertility remain poorly understood to a large extent. Endocrine-disrupting chemicals (EDCs) have been regarded as one of the factors associated with declining male fertility because of their xenobiotic nature, ubiquitous presence, and constant exposure through the skin, air, food, and drink (Gore et al., 2015; Sweeney et al., 2015; Sidorkiewicz et al., 2017; Segal and Giudice, 2019; Rodprasert et al., 2021). Environmental EDCs include pesticides, phthalates, and bisphenol A and their analogs, parabens, polychlorinated biphenyls, flame retardants, dioxins, solvents, and many others (Gore et al., 2015). The pyrethroid compounds represent an important class of pesticides used as insecticides with extensive applications for agricultural and residential purposes (Xu et al., 2008; Brander et al., 2016; Marettova et al., 2017; Solecki et al., 2017; EPA U.S., 2019; Sheikh and Beg, 2022) and are considered potential EDCs (Zamkowska et al., 2018). The past few decades have witnessed an enormous rise in global pyrethroid consumption (Feo et al., 2010; Ranson et al., 2011). The increased consumption is attributed mainly to the high insecticidal potential, slow pest resistance, and broad-spectrum application of pyrethroids. Furthermore, other advantages, such as less tissue accumulation, low human toxicity due to poor dermal absorption, swift metabolism, and less environmental persistence, contribute to increased pyrethroid consumption (Tang et al., 2018). As a result, organochlorine and organophosphorus pesticides were gradually phased out, which further amplified pyrethroid application in agricultural production and household use (Fenner et al., 2013; Saillenfait et al., 2016; Wang et al., 2016). The extensive use of pyrethroids resulted in a substantial increase in human exposure to pyrethroids from indoor and outdoor environmental sources (Burns and Pastoor, 2018). The two primary routes of pyrethroid exposure are diet and occupational exposure. The other routes of exposure include dermal contact and inhalation of contaminated household dust (ATSDR, 2003).

A thorough review of available epidemiological studies indicated an association between pyrethroid exposure and male infertility (Koureas et al., 2012; Saillenfait et al., 2015; Zamkowska et al., 2018; Castiello and Freire, 2021). For example, pyrethroid exposure was associated with male reproductive toxicity, and concerns regarding semen quality, sperm DNA, reproductive hormones, pregnancy outcomes, and developmental problems were raised (Saillenfait et al., 2015). Other studies also reported poor semen quality, such as low sperm count and abnormal sperm morphology in men exposed to pyrethroids (Perry et al., 2007; Hu et al., 2020). Nevertheless, the underlying molecular mechanisms for pyrethroid-induced male reproductive abnormalities are poorly understood. However, a critical review of the available literature suggested various possible molecular mechanisms for pyrethroid-induced male reproductive toxicity, such as steroid synthesis inhibition, inducing oxidative stress, acting as ER modulators, and antagonizing the AR (Wang et al., 2020). Furthermore, it was also proposed that pyrethroids cause reproductive abnormalities by interfering with the hypothalamic–pituitary–gonadal (HPG) axis, including reproductive hormone receptors (Zhang et al., 2008; Chrustek et al., 2018; Lu et al., 2019; Wang et al., 2020). In adult men, an inverse association was found between the urinary pyrethroid metabolites, serum inhibin B, testosterone, and free androgen index, along with a positive association with serum FSH and LH (Meeker et al., 2009). In addition, the anti-androgenic activity of several pyrethroids, including cypermethrin and permethrin, by antagonizing the androgen receptor (AR) has been reported (Zhang et al., 2008; Sheikh and Beg, 2022). A recent study reported that cypermethrin inhibited AR transcription by repressing the molecular interaction between the AR and activator proteins ARA70 and ARA55, subsequently contributing to male reproductive toxicity (Ding et al., 2020). Another study reported that cypermethrin displayed anti-androgenic effects by inhibiting dihydrotestosterone (DHT)-induced amino- and carboxyl-terminal interaction of the AR (Hu et al., 2012). Similarly, the anti-androgenic effects of cypermethrin by enhancing the associations of the AR with the corepressor silencing mediator for thyroid hormone receptors (SMRT) and nuclear receptor corepressors (NCoR) were also reported (Pan et al., 2013). The AR is an essential steroid nuclear receptor for male reproduction, which is activated following the binding of an androgenic hormone, such as testosterone or DHT (Roy et al., 1999; Lu et al., 2006). Testosterone represents a major circulating androgen synthesized from cholesterol and is converted into DHT (a metabolic product of testosterone), which is more active than testosterone (Sakkiah et al., 2018). The AR acts as a DNA-binding transcription factor regulating gene expression and plays a vital role in reproductive development in male people ((Mooradian et al., 1987; Zhang et al., 2008). The androgen signaling disruption by pyrethroids likely leads to abnormalities in male reproductive development, especially during reproductive differentiation and the development of male fetuses during early pregnancy. Therefore, in view of the crucial role of AR signaling in male reproductive development and the increasing pyrethroid exposure, this study was designed and executed to evaluate AR signaling disruption by commonly used pyrethroids.

All pyrethroids are categorized into two groups based on their physical properties and toxicity. The compounds included in these two groups are represented as class I and class II (Gajendiran and Abraham, 2018). Our previous study reported a structural binding study of the AR with permethrin, which belongs to class I pyrethroids (Zughaibi et al., 2022). However, in this study, we considered two commonly used class II compounds: cypermethrin and deltamethrin. Apparently, this is the first study focusing mainly on the structural binding characterization of class II compounds against the AR. In the present investigation, the ligands cypermethrin and deltamethrin were subjected to structural binding characterization by the molecular docking simulation approach against the AR ligand-binding pocket. The current study aimed to investigate the potential interference of these compounds in reproductive development by hindering androgen hormone binding to the AR. In addition, this study also provided insights into the structural binding characterization of cypermethrin and deltamethrin in the AR ligand-binding pocket.

## 2 Results

**FIGURE 1:**
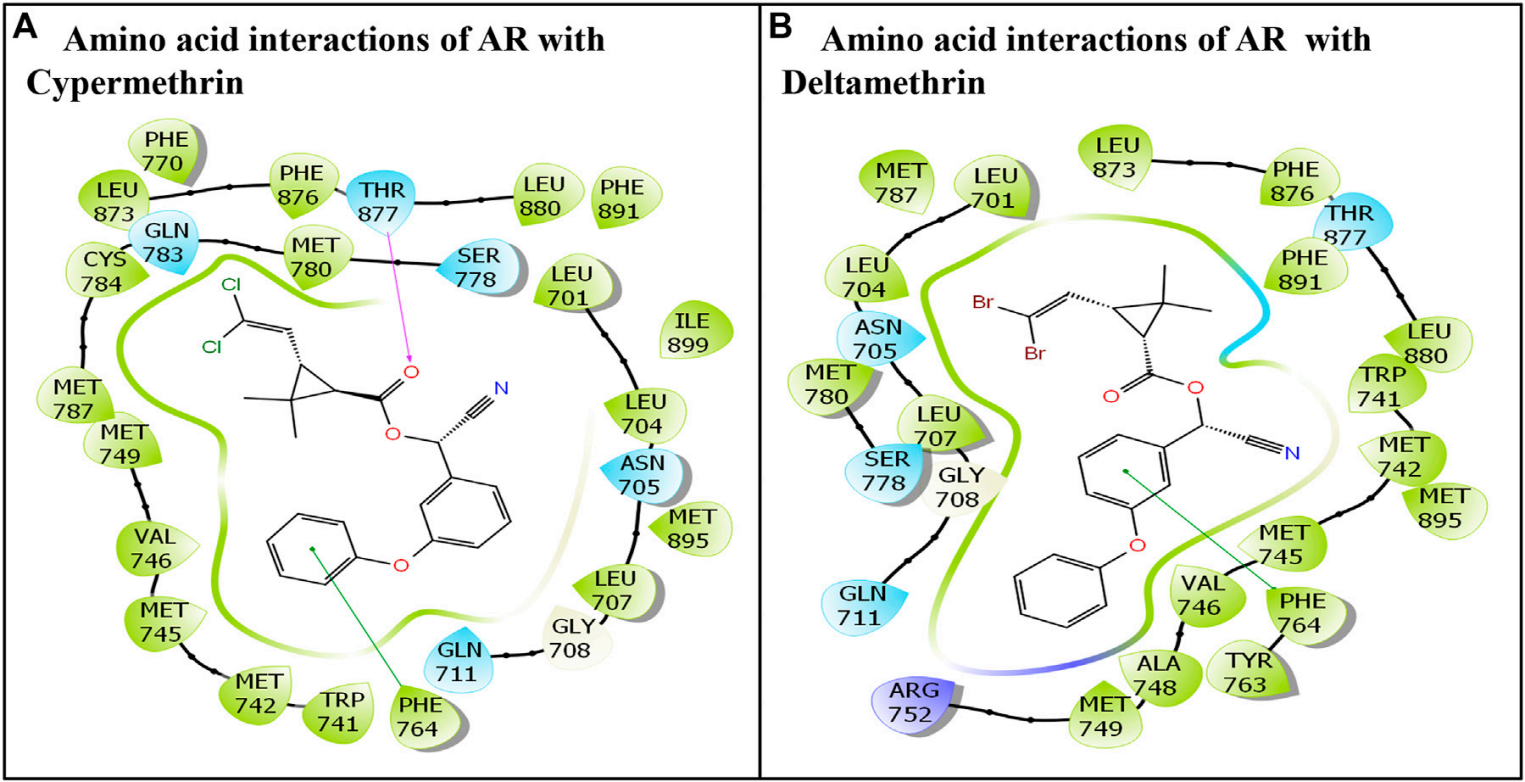
Molecular interactions of class II commonly used pyrethroids: **(A)** cypermethrin and **(B)** deltamethrin with residues lining AR ligand-binding pocket.

The commonly used class II pyrethroids—cypermethrin and deltamethrin—exhibited successful docking in the AR ligand-binding pocket. Both ligands cypermethrin and deltamethrin were placed stably in the AR ligand-binding pocket, following induced fit docking (IFD), indicating their firm binding. The IFD approach generated several docking poses for each ligand. However, only the best poses were chosen and carried forward for structural characterization. In addition, the AR native ligand, testosterone, was also placed stably in the AR ligand-binding pocket. Here, the best-ranking pose was also chosen for further analysis. All the chosen poses for both ligands exhibiting molecular interactions of amino acid residues with respective ligands are presented (Figure 1). Cypermethrin displayed interactions with 25 amino acid residues (Figure 1A), but deltamethrin displayed interactions with 24 amino acid residues of the AR (Figure 1B).

### 2.1 IFD of the cypermethrin ligand with AR

The cypermethrin–AR docking complex displayed several interactions with AR amino acid residues. Overall, 25 amino acid residues of the AR displayed various molecular interactions, such as hydrophobic, hydrogen bonding, and van der Waals interactions, with the ligand cypermethrin. The amino acid residues involved in various interactions were Leu-701, Leu-704, Asn-705, Leu-707, Gly-708, Gln-711, Trp-741, Met-742, Met-745, Val-746, Met-749, Phe-764, Phe-770, Ser-778, Met-780, Gln-783, Cys-784, Met-787, Leu-873, Phe-876, Thr-877, Leu-880, Phe-891, Met-895, and Ile-899. Moreover, the pi–pi interaction was displayed by Phe-764 (Figure 1A). In addition, one hydrogen bond interaction was also shown by Thr-877.

**FIGURE 2:**
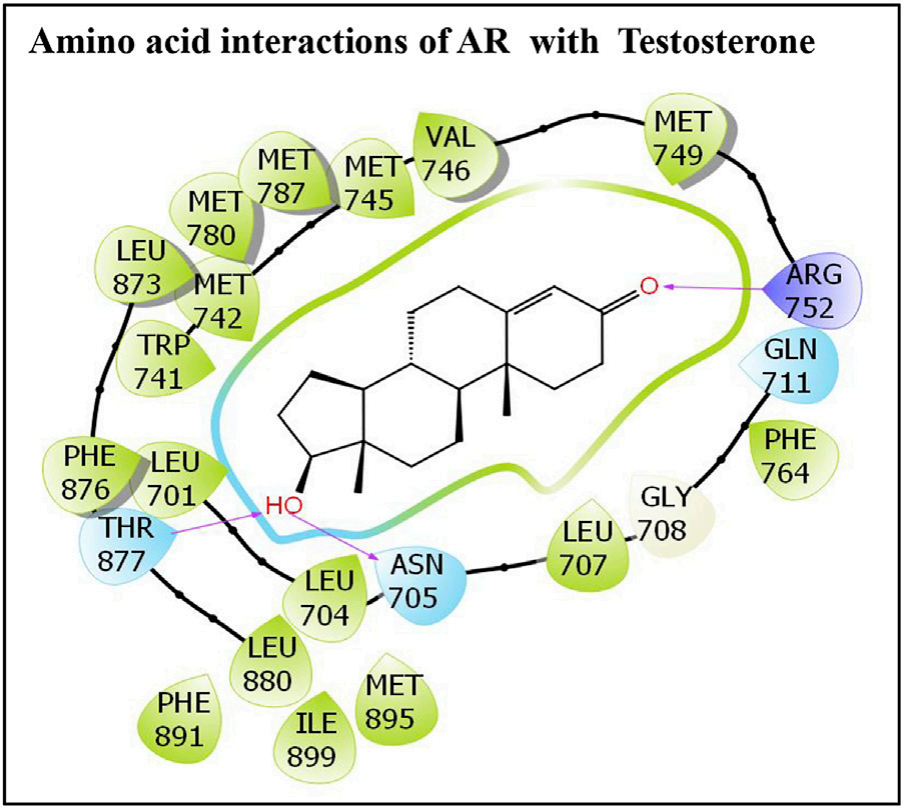
Molecular interactions of an AR native ligand, testosterone, with residues lining AR ligand-binding pocket.

Similarly, the molecular interaction of the AR native ligand, testosterone, with AR amino acid residues is also presented (Figure 2). Altogether, 22 AR amino acid residues displayed various molecular interactions with the AR native ligand, T, that is, Leu-707, Gly-708, Gln-711, Trp-741, Met-742, Met-745, Val-746, Met-749, Arg-752, Phe-764, Met-780, Met-787, Leu-873, Phe-876, Thr-877, Leu-880, Phe-891, Met-895, and Ile-899. In addition, three hydrogen bonding interactions, each by three amino acid residues, Asn-705, Arg-752, and Thr-877, with ligand testosterone, were observed (Figure 2).

The other parameters, such as IFD, Dock score, and Glide score, essential for structural binding analysis and characterization of cypermethrin and the AR native ligand, testosterone, are also presented (Table 1). In addition, another important parameter essential for analysis is the binding energy. The estimated binding energy values are also presented (Table 1). However, the estimated binding energy values for the AR native ligand, testosterone, and cypermethrin are very close. Moreover, the commonality among the AR interacting amino acid residues between the AR-native ligand and AR-cypermethrin docking complexes was 100%.

**TABLE 1:** Structural binding indices of class II pyrethroids, cypermethrin and deltamethrin, and AR native ligand, testosterone, against AR ligand-binding pocket.

Ligand	Number of interacting residues	Percentage of interacting residues common with native ligand (%)	IFD score	Docking score (Kcal/mol)	Glide score (Kcal/mol)	MMGB-SA (Kcal/mol)
Cypermethrin	25	100	−574.73	−10.75	−10.75	−138.39
Deltamethrin	24	95.45	−576.04	−11.23	−11.23	−137.49
Testosterone	22	100	−577.54	−12.87	−12.87	−152.82

### 2.2 IFD of deltamethrin ligand with AR

The docking display pose of deltamethrin exhibited 24 amino acid residues engaged in various molecular interactions with the AR (Figure 1B). Furthermore, the comparison between the docking poses of the AR native ligand, testosterone, and deltamethrin revealed approximately 96% overlap in amino acid interactions. However, several other molecular interactions were also observed in the deltamethrin–AR complex due to additional amino acid residues (i.e., Ala-748, Tyr-763, and Ser-778) (Figure 1B). In addition, Ile-899 residues present in the native ligand were missing in the deltamethrin–AR complex. Furthermore, one pi–pi interaction was also displayed by Phe-764.

## 3 Discussion

This study aimed to characterize the structural binding parameters, including the molecular interactions of commonly used class II pyrethroids—cypermethrin and deltamethrin—in the AR ligand-binding pocket. This study was performed to advance our understanding of the potential AR signaling disruption by the aforementioned ligands, which could have subsequent male infertility consequences. The in-depth result analysis of this study indicated the stable binding of both ligands (cypermethrin and deltamethrin) into the AR ligand-binding pocket. Furthermore, the stability and good quality of AR–ligand complexes were indicated by the estimated values of structural binding parameters, such as the IFD score, Glide score, Dock score, and binding energy. The several molecular interactions displayed in the AR–ligand complexes, such as hydrogen bond, pi–pi interactions, and salt bridge, contribute significantly to the stability of these complexes. The critical analysis of the comparison of the AR native ligand’s (testosterone) docking pose with the best-chosen docking pose of cypermethrin and deltamethrin indicated 80%–90% commonality in the interacting amino acid residue lining the AR ligand-binding pocket. Furthermore, the binding energy values calculated for cypermethrin and deltamethrin were also similar to the AR native ligand, testosterone. The values calculated for cypermethrin and deltamethrin were close to the values calculated for the AR native ligand, testosterone. Therefore, the results of this study suggest the potential for these ligands to disrupt the AR signaling, which might subsequently have an adverse impact on male reproductive development and fertility.

Previous *in silico* studies on the structural binding characterization of cypermethrin and deltamethrin against the AR are unavailable. However, we recently reported the potential AR signaling disruption by one of the commonly used pyrethroid compounds, which is permethrin (Zughaibi et al., 2022). Nevertheless, several *in vitro* and epidemiological studies conducted on various pyrethroids, including cypermethrin and deltamethrin, are consistent with our findings and have reported their antagonizing action on AR, hence displaying anti-androgenic activities (Kojima et al., 2004; Kim et al., 2006; Sun et al., 2007; Xu et al., 2008; Tange et al., 2014). Furthermore, our findings are supported by previous reports, which indicated HPG axis disruption by pyrethroids, resulting in abnormal male reproductive hormone levels, such as increased sex hormone-binding globulin and a decrease in the free androgen index (Ye and Liu, 2019). The fact that AR signaling is an important pathway of the HPG axis could make it a potential target of pyrethroids, subsequently disturbing the HPG axis and hampering male reproductive developmental functions (Jin and Yang, 2014; Plant, 2015). In addition, the estrogenic or anti-estrogenic activity of various pyrethroid compounds, including cypermethrin and deltamethrin, was also reported by various studies. They compete with the binding of estradiol to estrogen receptors and induce cell proliferation (Go et al., 1999; Chen et al., 2002; Kim et al., 2004; Zhao et al., 2008).

Numerous epidemiological association studies indicated the adverse impact of pyrethroid exposure on reproductive health. Pyrethroid exposure in male greenhouse workers was associated with reduced fecundability, suggesting an association between decreased fertility and pyrethroid exposure in men (Sallmén et al., 2003). Environmental and occupational exposure to pesticides, including the pyrethroids reviewed for the years 2012–2022, revealed diminished semen parameters, such as sperm concentration, sperm motility, sperm morphology, and sperm DNA integrity, as a consequence of pyrethroid exposure (Knapke et al., 2022). Another study reported an association between high fenvalerate exposure and abnormal semen quality, including decreased sperm motion parameters, sperm progression, beat cross frequency, and an increase in the abnormality rate of viscidity and coagulation (Lifeng et al., 2006). In this regard, a positive association between poor semen quality and pyrethroid exposure level was reported in 42 Japanese male partners of couples attending consultation in an infertility center (Toshima et al., 2012). In several other studies, a negative association between urinary pyrethroid metabolites and semen quality, including a decrease in sperm count, abnormal sperm cell morphology, and reduced testosterone levels in men, was reported (Meeker et al., 2008; Xia et al., 2008; Radwan et al., 2014; Hu et al., 2020). In addition to abnormal semen parameters, pyrethroid metabolite levels in urine in several ethnic populations, such as American and Chinese, were negatively associated with serum testosterone and inhibin B levels and positively associated with serum gonadotropin levels (Han et al., 2008; Meeker et al., 2009; Radwan et al., 2014). Conversely, a report of no association between serum hormone levels and urinary pyrethroid metabolite 3-PBA in 322 Japanese male students is also available (Yoshinaga et al., 2014). In another study on 240 healthy male participants, a significant positive correlation between the urinary pyrethroid metabolite 3-PBA levels and sperm DNA fragmentation was reported (Ji et al., 2011). Furthermore, urinary pyrethroid metabolites showed a negative association with the Y:X sperm chromosome ratio (Jurewicz et al., 2015; Jurewicz et al., 2016). A study conducted on the agricultural population of the southern region of Brazil reported that the recent application of lambda-cyhalothrin was related to the increase in LH hormone levels in the male population, raising concerns about reproductive health (Santos et al., 2019). An *in vitro* study conducted on 20 normozoospermic semen samples indicated altered sperm cell function and DNA damage by cypermethrin (Zalata et al., 2014). Another study conducted on 19 fenvalerate-exposed workers revealed breaks in sperm DNA using Comet and TUNEL assays (Xia et al., 2004).

Similarly, several association studies on animals indicated the negative outcomes of pyrethroid exposure on reproductive health. For example, a systematic review and a meta-analysis on pyrethroid exposure in rodents indicated male reproductive system toxicity (Zhang et al., 2021). Furthermore, animal studies on rats reported an association between pyrethroid exposures and decreased levels of follicle-stimulating hormone (FSH), luteinizing hormone (LH), and testosterone. Furthermore, the association was also observed with epididymis and testis weight (Navarrete-Meneses and Perez-Vera, 2019). This study is of significant importance in determining the effect of commonly used pyrethroids on fertility outcomes in male people exploring infertility treatment. This study investigates the structural binding characterization and molecular interactions of cypermethrin and deltamethrin with AR leading to anti-androgenic effects and disturbing AR signaling. However, this is a simulation study and has its own limitations as well. Thus, more studies using *in vivo* and *in vitro* models are warranted to further confirm the results of this study. More specifically, an integrated multi-omics approach would provide a significant contribution in this regard, addressing the male infertility problems arising due to pyrethroid exposure.

## 4 Conclusion

This study aimed to explore the structural interactions of commonly used class II pyrethroids—cypermethrin and deltamethrin—with AR for potential disruption activity, which subsequently could lead to male infertility. The structural binding parameters for AR docking complexes with cypermethrin and deltamethrin indicated similarity with that of the AR native ligand docking complex (AR–testosterone), thus forming a stable and successful docking complex. In addition, the results also revealed high binding energy for both pyrethroids against the AR ligand-binding pocket, which was similar to the values calculated for the AR native ligand, testosterone. Overall, the results of the present study suggested that both the indicated ligands (cypermethrin and deltamethrin) have the potential to disrupt the AR signaling function and might subsequently affect the male reproductive functions, causing infertility.

## 5 Material and methods

The commonly used class II pyrethroids—cypermethrin and deltamethrin—were chosen, and their three-dimensional structural coordinates were downloaded from the PubChem compound database (https://pubchem.ncbi.nlm.nih.gov/). It was followed by structural binding characterization of these ligands using the Schrodinger 2017 suite with Maestro 11.4 as a graphical user interface (Schrodinger, LLC, New York, NY, 2017). The detailed methodology is described in our previous study (Sheikh, 2016; Zughaibi et al., 2022).

### 5.1 Protein preparation

The three-dimensional structural coordinates solved at 1.64 Å resolution for the crystal complex of the ligand, testosterone, with the AR (PDB code: 2AM9) were retrieved from Protein Data Bank (PDB) (http://www.rcsb.org/). The retrieved coordinates were imported to Glide docking software, and the protein crystal complex was subjected to further processing and prepared for docking studies using the protein preparation wizard workflow of Schrodinger Glide (Schrodinger suite 2017-4; Schrodinger, LLC), as described in our previous study (Sheikh, 2016; Zughaibi et al., 2022). Briefly, the missing hydrogen atoms and charges were added, and water molecules were removed from the crystal complex structure. It was followed by hydrogen bond network optimization and energy minimization.

### 5.2 Ligand preparation

**FIGURE 3:**
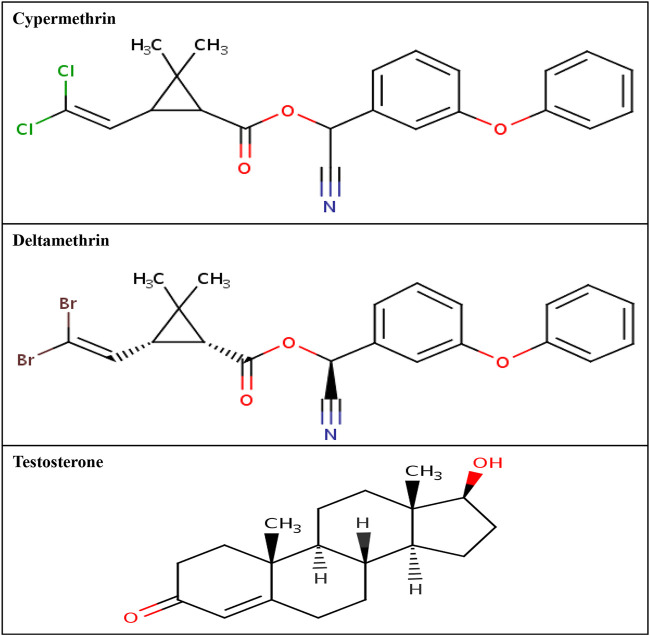
Two-dimensional structure of cypermethrin, deltamethrin, and AR native ligand, testosterone.

The three-dimensional structural coordinates for the commonly used class II pyrethroids (cypermethrin and deltamethrin) were downloaded from the PubChem compound database (https://pubchem.ncbi.nlm.nih.gov/). The PubChem compound identity for the ligand cypermethrin is 2912, whereas the PubChem compound identity for deltamethrin is 40585. These ligands were processed and prepared for simulation studies using the LigPrep module of Schrodinger (Schrodinger 2017: LigPrep, Schrodinger, LLC). The two-dimensional structures of cypermethrin and deltamethrin are presented in Figure 3.

### 5.3 IFD

The Schrodinger’s IFD module was employed to perform the docking of the AR native ligand, testosterone, and class II pyrethroids—cypermethrin and deltamethrin—in the AR ligand-binding pocket, as described in detail in our previous study (Sheikh, 2016; Zughaibi et al., 2022). Briefly, first, a grid was generated at the binding site of the AR native ligand, testosterone. It was followed by constrained minimization of the AR using the protein preparation step. The IFD induces flexibility in both the ligand and ligand-binding pocket of protein receptors and does not adopt a rigid docking approach. First, initial Glide docking was performed using a softened potential and optional side chain removal for all the ligands, and by default, twenty docking poses were retained. The side chains in amino acids were predicted, followed by energy minimization for the receptor and ligand in each pose. It was followed by Glide re-docking and IFD score estimation. Likewise, an extended sampling protocol was also performed. Similarly, the IFD was also performed on the AR native ligand, testosterone.

### 5.4 Binding affinity calculations

The binding affinity of cypermethrin and deltamethrin for the AR ligand-binding pocket was estimated using the molecular mechanics generalized Born surface area (MMGB-SA) function in the Prime module of Schrodinger 2017, as described previously (Sheikh, 2016; Zughaibi et al., 2022).

## Data Availability

The original contributions presented in the study are included in the article/Supplementary Material. Further inquiries can be directed to the corresponding author.
